# Microbe-Induced Inflammatory Signals Triggering Acquired Bone Marrow Failure Syndromes

**DOI:** 10.3389/fimmu.2017.00186

**Published:** 2017-02-24

**Authors:** J. Luis Espinoza, Ritesh Kotecha, Shinji Nakao

**Affiliations:** ^1^Department of Hematology and Oncology, Graduate School of Medical Science, Kanazawa University, Kanazawa, Ishikawa, Japan; ^2^Department of Medicine, Beth Israel Deaconess Medical Center, Boston, MA, USA

**Keywords:** bone marrow failure syndromes, aplastic anemia, virus-induced anemia, microbioma, microbe immunity

## Abstract

Acquired bone marrow failure syndromes encompass a unique set of disorders characterized by a reduction in the effective production of mature cells by the bone marrow (BM). In the majority of cases, these syndromes are the result of the immune-mediated destruction of hematopoietic stem cells or their progenitors at various stages of differentiation. Microbial infection has also been associated with hematopoietic stem cell injury and may lead to associated transient or persistent BM failure, and recent evidence has highlighted the potential impact of commensal microbes and their metabolites on hematopoiesis. We summarize the interactions between microorganisms and the host immune system and emphasize how they may impact the development of acquired BM failure.

## Introduction

Bone marrow failure syndromes (BMFS) are a group of heterogeneous disorders defined by the loss or malfunction of hematopoietic stem cells (HSCs). Deficient cell production can be seen across multiple lineages, resulting in a loss of erythrocytes, granulocytes, or platelets. Distinct syndromes are, therefore, defined by the specific cells affected and include pure red cell aplasia (PRCA), amegakaryocytic thrombocytopenic purpura, aplastic anemia (AA), and myelodysplastic syndrome. AA, the paradigm BMFS, is characterized by a deficiency of HSCs resulting in peripheral pancytopenia and hypoplastic bone marrow (BM) (1, 2). This may occur as the result of inherited abnormalities as seen in syndromes like Fanconi anemia, dyskeratosis congenital, and Shwachman–Diamond syndrome, or may be an acquired phenomena (3).

The primary mechanism of acquired AA centers on the immune-mediated destruction of HSCs, and highly immunosuppressive therapies provide excellent and durable clinical responses (4). Several immune cell abnormalities are also commonly found in patients, including dysregulated CD4+, CD8+, and Th-17 T-cell responses, as well as reduced numbers of regulatory T-cells. Furthermore, many patients have elevated circulating levels of inflammatory or myelosuppressive cytokines like interferon (IFN)-γ, tumor necrosis factor alpha (TNF)-α, and transforming growth factor beta (TGF-β) (5).

Despite efforts that have revealed circulating autoantibodies in acquired AA patients (6–8), the identification of autoantigens able to elicit cytotoxic T-cell responses and breach immune tolerance leading to the destruction of HSCs has been difficult. Current theories suggest that, similar to other autoimmune diseases, the initial immune response may be triggered by drugs, chemicals, or pathogens, or through the generation of neoantigens *via* epigenetic mechanisms (4, 5, 9, 10). Interestingly, autoimmune illnesses like rheumatoid arthritis, systemic lupus erythematous, or ulcerative colitis sometimes precede the development of acquired AA (11–13). As transient and persistent BM hypoplasia have been linked to various microorganisms (14), dysbiosis between the gut microbiota and immune system may serve as an initial insult in the development of BMFS (11).

We herein report an overview of the complex interplay between microorganisms, the immune system, and hematopoiesis and discuss the implications these interactions may have in the pathogenesis of acquired BMFS.

## Regulation of Hematopoiesis by Inflammatory Signals

Interplay between HSCs and their microenvironment determines whether or not these cells will undergo differentiation, proliferation, or apoptosis. Secreted factors like erythropoietin, thrombopoietin, IL-3, GM-CSF, and stem cell factor (SCF) positively regulate the HSC maintenance and differentiation during steady-state hematopoiesis (1–3). Surrounding mesenchymal stem cells (MSCs) and other BM niche components support HSCs and ensure their stem cell phenotype through the release of TGF-β, SCF, CXCL12, and angiopoietin-1 (15).

In response to systemic injury, HSCs are signaled to proliferate and differentiate. HSCs express cytokine, chemokine, and pathogen recognition receptors (PRRs) and can be directly triggered by activated immune effector cells, pathogens, or by surrounding stem cells (16, 17). In bacterial infections, the rapid consumption of granulocytes triggers HSCs to proliferate along the myeloid lineage (15, 18). In contrast, viral infections mainly involve IFN-α and IFN-β signaling. Type I IFNs prevent viral replication and induce HSCs to transiently proliferate, whereas persistent type I IFN signaling may lead to HSC exhaustion (19, 20).

Interferon-γ secreted by activated T-cells and NK cells modulates hematopoiesis differentially based on acute on chronic signaling. For instance, HSCs have been shown to enter active cell cycle stages and differentiate in mice treated with IFN-γ. However, chronic IFN-γ stimulation impairs the function of HSCs, leading to the development of cytopenia (21, 22).

Other signaling molecules released during systemic stress may also impact hematopoiesis. TNF-α produced by CD8+ T-cells enhances HSC clonogenicity and prevents HSC apoptosis both *in vitro* and *in vivo* (23). IL-6, a pleiotropic cytokine secreted by BM stromal fibroblasts, leads to the expansion of myeloid progenitors and blocks the development of erythroid cells (24). In response to microbial infection, additional cytokines like IL-1, IL-17, and IL-27 may influence blood cell development, particularly through the induction of HSC expansion and granulopoiesis (20, 25).

Many inflammatory signals maintain immune homeostasis and transiently stimulate hematopoiesis in the promotion of the host defenses during stress. However, the prolonged stimulation of HSCs may induce an opposing effect leading to anergy, chronic exhaustion, and apoptosis. Cytopenias associated with chronic inflammatory conditions and autoimmune diseases, therefore, likely stem from the sustained failure of HSC renewal and differentiation (17, 26, 27).

## Microbiota and Microbial Metabolites Shape Hematopoiesis

The complex system of bacteria, viruses, and fungi living in the human body is referred to as the microbiota. These commensal organisms colonize multiple body niches, with colonic microorganisms being the most abundant (28–30).

The microbiome and its associated metabolites have recently been functionally linked to hematopoiesis, as evidence suggests that the BM myeloid population strongly correlates with microflora complexity. In germ-free mice, the granulocyte and monocyte populations, but not the lymphoid progenitor populations, increased with greater gut flora complexity (31). A lower microbiota diversity was also associated with an overall worse survival and transplant-related mortality in patients receiving allogenic stem cell transplantation (32). Additionally, germ-free and antibiotic-treated mice have impaired functional clearance of systemic bacterial infections. Therefore, many have proposed that commensal microbes play a significant role in HSC maintenance and alterations, and the absence of gut microflora may lead to detrimental downstream defects in immunity (33, 34).

Substances like dietary fiber may exert indirect effects on hematopoiesis through shaping microbial composition. For instance, mice given a fiber-rich diet have alterations in *Firmicutes, Bacteroidetes*, and *Bifidobacteriaceae* populations. These microbes metabolize fiber to short-chain fatty acids (SCFAs), and mice treated with SCFA have larger populations of macrophages and dendritic cell precursors in the BM (35). In this model, high-fiber diet mice had increased circulating SCFAs and were found to have protection against allergic inflammatory lung diseases compared to low-fiber diet, low-level circulating SCFA animals (35).

Pathogen recognition receptors found on HSCs, including toll-like receptors 2, 3, 4, 7, and 9, enable HSCs to recognize and respond to various pathogen-derived products (36, 37). Quiescent HSCs are activated upon acute exposure to these pathogens or products and in turn proliferate. In contrast, chronic exposure to systemic TLR ligands appears to have myelosuppressive effects, as supported by HSC exhaustion seen in mice exposed to repeated administrations of low-dose LPS for 6 weeks (38). Frequent gut microbe translocation, coupled with persistent, detectable serum LPS found in HIV infection has been proposed as mechanism for HIV-related myelosuppression (36). Furthermore, TLR4 may be activated by fatty acids and high levels of circulating metabolites, as found in patients with chronic metabolic syndrome (39). Kell and Pretorius have also proposed that bacterial translocation from dormant bacterial reservoirs provide a persistent source of low-grade inflammation *via* immune-mediated signals triggered by LPS and other pathogen-associated molecular patterns (PAMPs) (40). This supports the strong association between altered gut microbiota and various autoimmune diseases and may underscore the frequent association of ulcerative colitis, a disease characterized by high bacterial leakage, with BMFS (11).

**Figure 1:**
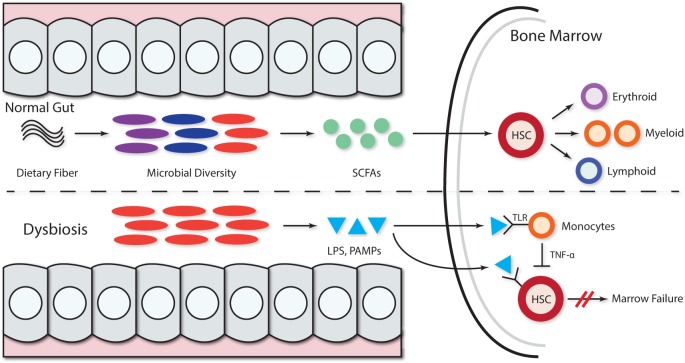
**Microbiota and microbial metabolites can shape hematopoiesis and the immune response**. Commensal microbes promote the maintenance of both hematopoietic stem cells (HSCs) and precursor myeloid cells. The absence of commensal microbes leads to defects in several innate immune cell populations, including neutrophils, monocytes, and macrophages. Feeding mice a diet rich in fiber changed the ratio of *Firmicutes* to *Bacteroidetes* and *Bifidobacteriaceae*. The presence of a complex intestinal microbiota specifically amplifies myelopoiesis in the bone marrow (BM). Dietary fiber is metabolized by gut microbiota, thereby increasing the levels of circulating short-chain fatty acids (SCFAs) and promoting the growing of myeloid precursors without affecting lymphoid progenitors in the BM. In the context of dysbiosis, the growth of pathogenic microbes acts as dormant bacterial reservoir that provides a source of persistent low-grade inflammation mediated by LPS and other pathogen-associated molecular patterns (PAMPs) that persistently stimulate hematopoietic stem progenitor cells *via* pathogen recognition receptors like TLR (TLR4, TLR7, and TLR9), leading to hematopoiesis inhibition. The LPS stimulation of TLR monocytes induces TNF-alpha secretion, and this persistent stimulation of HPSCs may further inhibit hematopoiesis *via* exhaustion.

The association between alterations in the gut microbiome and BMFS has not been systematically investigated. However, evidence for the pivotal role of gut flora in immune system priming, education, and regulation (41, 42) suggests that microbiota, their metabolic products, or PAMPs can lead to the development of hematological disorders (34). In patients with acquired BMFS, microbes and their metabolites may inhibit hematopoiesis and enact negative downstream effects on HSCs (Figure 1). However, the details of the mechanisms have yet to be elucidated.

## BM Failure Induced by Microbial Infection

Several microbial infections have been linked to the development of acquired BMFS. The mechanisms underlying how pathogens induce hematopoietic dysfunction are poorly understood for most diseases, except parvovirus B19 infection-related aplasia (43). Many hypotheses for disease pathogenesis center on the direct infection of HSCs, viral recognition by HSCs *via* PRRs, inflammation-mediated effects by surrounding cells or response to changes in the stem cell microenvironment (15, 19, 20, 44). Cytomegalovirus (CMV), parvovirus B19, and the Epstein–Barr virus (EBV) induce HSC injury through direct toxic effects from pathogens (14, 45). However, the majority of pathogen-related cases are thought to be due to the excessive activation of immune effector cells, leading to an overwhelming release of myelosuppressive cytokines and negative proliferation signaling to HSCs (Table 1).

**Table 1:** **Microbes triggering bone marrow (BM) failure**.

Microbe	Effects on hematopoiesis	Mechanism(s)	Target cells	Reference
**Virus**				
Parvovirus B19	Various cytopenias	Apoptosis of target cells	Erythroid progenitor	(43, 47–50)
	Anemia	Excessive inflammatory signals IL1-β, IL6, tumor necrosis factor-α, and interferon (IFN)-γ		
	Pure red cells aplasia			
	Aplastic anemia (AA)			
	Thrombocytopenic Purpura			

Epstein–Barr virus	Thrombocytopenia	Excessive inflammatory signals: TNF-α and IFN-γ	HPSC	(54–58)
	AA	HPSC inhibition by virus-specific T-cells	T-cells	
	Pure red cells aplasia			

Dengue virus	Leukopenia	Apoptosis of progenitor cells	Hematopoietic stem progenitor cells, megakaryocyte progenitor	(62–65, 69, 70)
	Thrombocytopenia	Excessive inflammatory signal: multiple cytokines		
	Severe AA			

HAAA	AA	Excessive inflammatory signals	Indirectly HPSC?	(74–76)
		T-cell activation		
		Multiple cytokines		

Cytomegalovirus	AA	Stromal function failure	Mesenchymal stem cells	(77, 78)
	Anemia			

Human herpes virus-6	Anemia	Apoptosis of target cells?	Granulocyte macrophage	(79, 80)
	Pancytopenia		Megakaryocyte progenitors	

HIV	Anemia	Excessive growth of bacterial	HPSC	(36, 40)
		Sustained activation of pathogen recognition receptors (PRRs), TLRs by LPS or other pathogen-associated molecular patterns (PAMPs)		

**Bacteria**				
*Anaplasma phagocytophilum*	Pancytopenia	Excessive inflammatory signals	Circulating granulocyte	(85–88)
		Myelosuppressive cytokines		

*Ehrlichia chaffeensis*	Pancytopenia		Granulocyte	(89, 90)
	Anemia			
	Thrombocytopenia			

Tuberculosis	Pancytopenia	Granuloma infiltration in BM	BM niche	(91–93)
		Maturation arrest?		
		Hypersplenism?	HPSC?	
		Histiocytic hyperplasia?		

Dysbiosis	Anemia?	Persistent release of PAMPs?	HPSC?	(11, 34, 36, 38, 40)
	AA?	Sustained stimulation of HPSCs *via* PRRs?		

## Parvovirus B19-Induced BM Failure

Human parvovirus B19 (B19V) is a small DNA erythrovirus associated classically with fifth disease or erythema infectiosum (46). Primarily spread *via* respiratory droplets, the virus targets erythroid progenitors *in vivo* (43). *In vitro*, the virus is propagated in primary erythroid progenitors BFU-E and CFU-E, and its replication is enhanced under hypoxic conditions. B19V also induces apoptosis in erythroid progenitors in the BM, resulting in hypoplasia (43). In an immunocompromised host, persistent B19V infection can cause chronic anemia, aplastic crisis, PRCA, and idiopathic thrombocytopenic purpura (47, 48).

B19V cytotoxicity appears to be mediated *via* the NS1 protein, which in turn activates caspase 3, 6, and 8, increasing the erythroid cell sensitivity to apoptosis induced by TNF-α (49). In addition, B19V infection is associated with the systemic activation of monocytes, T-cells, and NK cells and correlates with an elevation in serum inflammatory cytokines IL-1β, IL-6, TNF-α, and IFN-γ (43). B19V infection has also been implicated in autoimmunity, as some patients may develop antibodies, including antinuclear, antiphospholipid, anti-smooth muscle, gastric parietal cell antibodies, and rheumatoid factors (49). Apoptotic bodies generated during B19V infection contain many different self-antigens and may serve as a reservoir of autoimmunity priming during infection (50).

## EBV-Induced BM Failure

Epstein–Barr virus remains one of the most common viruses afflicting humans, most frequently causing infectious mononucleosis. EBV-infected cells are also associated with cell transformation and several malignancies, including Hodgkin’s lymphoma, Burkitt’s lymphoma, nasopharyngeal carcinoma, gastric cancer, and HIV-associated neoplasms, such as hairy cell leukoplakia (51, 52). EBV has also been associated with an increased risk for autoimmune disorders, including rheumatoid arthritis, dermatomyositis, and systemic lupus erythematosus (53).

In immunocompromised patients, EBV can be associated with a wide range of hematopoietic effects, including BMF and lymphoproliferative disease (51). Single cell lineage disorders like thrombocytopenia with ITP-like syndrome (54) or PRCA (55) have been found in some patients, while others have shown pancytopenia mimicking acquired AA (56). EBV-induced aplasia likely involves excessive immune activation, as experimental data have shown that activated T-cells exposed to autologous EBV-infected B-cells inhibit HSC growth (57). Clinically, patients with EBV-induced acquired AA may respond well to immunosuppressive therapy, and some suggest it may play a role in idiopathic acquired AA cases (58).

## Dengue Virus (DENV)-Induced BM Failure

Five distinct serotypes of DENV, a single-stranded RNA arbovirus, has been identified, and all cause dengue fever. Typically, DENV infection induces multiple hematologic abnormalities, including leukopenia, neutropenia, and thrombocytopenia (59). BM biopsies isolated during DENV infection are characterized by abnormal megakaryopoiesis, reticulocytopenia, and granulocytopenia (60, 61).

Although the pathophysiology of DENV-induced BM failure is not well understood, accumulating evidence indicates a combination of an excessive immune response and viral infection of progenitor cells (62, 63). During the acute phase of infection, DENV infects and proliferates in HSC progenitors and CD61+ megakaryocyte progenitor cells (60, 64), thereby inducing transient BMF (63). In addition, DENV infection is associated with the activation of several innate immune responses, including IFN α/β, MIP-1α/β, viperin, and CXCL-10 release, which may inhibit hematopoiesis (65–68). DENV infection has also been shown to preferentially induce production of IFN type III (IFN-λ1) from human dendritic cells, signaling through TLR-3 (69). Similar to other interferons, IFN-λ1 acts as a myelosuppressive factor (70). As the severity of hematological dysfunction can be quite variable and clinical responses are usually achieved *via* immunosuppression, efforts aimed at characterizing autoimmune responses during both subclinical and clinically significant infections are needed (62, 63).

## Hepatitis-Associated BM Failure (HABMF)

Hepatitis-associated BM failure is a distinct variant usually seen 2 or 3 months following an episode of acute hepatitis (71). Although in some cases this entity has been reported in association with hepatitis A, B, C, E, and G viral infections (71, 72), as well as parvovirus B19, EBV and CMV, most patients with HABMF are negative for all known viruses (71). HABMF can be self-limited but often is severe and even fulminant (71, 72); however, the severity appears to be independent of the age, sex, or severity of hepatitis (73). Typically, both the hematologic abnormalities and liver function parameters improve with immunosuppressive therapies (74). When HABMF manifests as severe AA, it represents a life-threatening condition that requires urgent hematological therapy with supportive care and stem cells transplantation (72). Several immunological abnormalities have been documented in patients with HABMF, including increased soluble IL-2 receptor, low ratios of CD4+/CD8+ cells, high percentages of CD8+ cells, and reduced proportions of CD4+CD25+ regulatory T-cells (74, 75). Notably, clonal expansion of T-cells with conserved antigen specificity has been found in HABMF patients (76), suggesting that abnormal immune responses underlie the disease and viral antigens may elicit T-cell responses that cross-react with antigens expressed by HSCs.

## Other Virus-Related BM Failure

Other viruses can also induce BMFS *via* similar mechanisms in many patients. For instance, several cases of CMV-associated BMFS have been documented, and experimental data have shown CMV infection and replication in MSCs, along with an impaired stromal function (77, 78). Anecdotic associations between human herpes virus 6 with BMFS, mainly in the posttransplantation setting, have been also reported (79) and appear to be related to the direct viral injury of granulocytes, macrophages, and megakaryocyte progenitors *in vitro* (80). Interestingly, respiratory syncytial virus has also been shown to infect and replicate in human BM stromal cells (81), although its association with resultant BMF does not appear to be common.

## Marrow Aplasia and Bacterial Infections

Bacterial infection of HSCs is uncommon, as these cells are rare, quiescent and reside in a protected microenvironment with surrounding MSCs. Stromal elements are capable of inhibiting the growth of several Gram-negative and Gram-positive bacteria (82). Additionally, *in vitro* experiments have suggested that HSCs may be resistant to intracellular bacteria like *Listeria monocytogenes, Salmonella enterica*, and *Yersinia enterocolitica* (83). These findings are consistent with clinical observations that bacterial pathogens are rarely associated with direct hematopoietic dysfunction. Notably, human CD34^+^ hematopoietic stem progenitor cells exposed to *Escherichia coli in vitro* produce pro-inflammatory cytokines, such as IL-1, IL-6, IL-8, and TNF-α, *via* NFκB activation (84), although the implications of these observations in the clinical setting are unknown.

One of the best characterized myelosuppressive pathogens is *Anaplasma phagocytophilum*, which causes granulocytic anaplasmosis or ehrlichiosis (85). This Gram-negative bacterium infects granulocytes and persists primarily within circulating granulocytes (86, 87), and infection typically results in multiple cytopenias, including anemia, leucopenia, and thrombocytopenia (86). Mouse models of infection show profound and rapid multilineage deficits in proliferation and differentiation, including B-cell depletion, erythroid depletion, granulocytic hyperplasia, and a significant downregulation of CXCL12 in the BM. These defects are accompanied by induction of myelosuppressive cytokine release such as MCP-1, MIP-2, TNF-α, and IL-6. The absence of infectious particles in the BM compartment suggests that hematopoietic suppression stems from the systemic activation of inflammatory signaling rather than direct infection (88).

*Ehrlichia chaffeensis* causing monocyte ehrlichiosis is also associated with the development of multiple cytopenias (89). Mouse models have supported the notion that microbial infection may lead to anemia, thrombocytopenia, and BM hypocellularity. Furthermore, in this model, the number of committed progenitors, including erythroid, granulocyte, and monocyte progenitors, in the BM was significantly fewer than in control mice (90).

Finally, pancytopenia with BM suppression is an uncommon hematological manifestation of active tuberculosis (91). This may be due to granulomatous inflammation and focal necrosis in the BM (92). Although not clearly defined, other mechanisms that may lead to pancytopenia in these patients include histiocytic hyperplasia, HSC maturation arrest, and hypersplenism (92, 93).

## Concluding Remarks and Future Directions

During acute infections, the immune system regulates the expansion and differentiation of HSCs in an attempt to appropriately combat invasive pathogens. However, sustained signaling mechanisms may lead to chronic HSC exhaustion and BM suppression. Clinically, many microbial infections have been associated with BMFS; however, identifying patients who are susceptible to hematopoietic suppression remains impossible at present. Immune-mediated BMF following clearance of viral infections may be a common mechanism; however, further investigation regarding inflammatory-related genes, immune education, and tolerance is needed. For instance, regulatory T-cells, which maintain self-tolerance and prevent excessive immune activation, have also been found to suppress colony formation *in vitro* and HSC myeloid differentiation *in vivo* (94, 95). Notably, the numbers of regulatory T-cells are significantly diminished in patients with acquired AA (96). Specific regulatory T-cell changes following microbial infections in certain system compartments have not been investigated, but may now be detectable with the advent of new cellular subset population analyses (97).

How chronic infections may affect HSCs over time remains unknown as well. As this review highlights, the gut microbiome may have a direct role in normal hematopoiesis, as microbes and their metabolic products may have downstream consequences. As proposed, dysbiosis may trigger autoimmune effects *via* the enzymatic posttranslation modification of proteins and generation of neoantigens for unique T-cell responses (98). This model is quite well-suited for studying the basis for immune-mediated BMFS, particularly in patients with known microbial alterations like inflammatory bowel diseases. However, further research that characterizes the microflora patterns, whether by genomic or metabolic recognition, may have novel diagnostic and prognostic utility. Interventions targeting the suppression and removal of distinct microbial species may have a tremendous impact on human health and disease.

## Author Contributions

JE and RK: literature search, wrote the manuscript, and designed Figure 1. SN wrote the manuscript.

## Conflict of Interest Statement

The authors declare that the research was conducted in the absence of any commercial or financial relationships that could be construed as a potential conflict of interest.
